# Recent Advances and Challenges in the Treatment of Advanced Pancreatic Cancer: An Update on Completed and Ongoing Clinical Trials

**DOI:** 10.3390/cancers17081319

**Published:** 2025-04-14

**Authors:** Abhinav Shenoy, Amar Yousif, Muhammad Delwar Hussain

**Affiliations:** 1 College of Engineering, Texas A&M University, College Station, TX 77843, USA; abhinavshenoy001@tamu.edu; 2 Department of Pharmaceutical Sciences, School of Pharmacy and Health Professions, University of Maryland Eastern Shore, Princess Anne, MD 21853, USA; aeyousif@umes.edu

**Keywords:** advanced pancreatic cancer, targeted therapy, molecular profiling, genetic mechanisms, selective inhibitors, standalone treatments

## Abstract

Pancreatic cancer is a deadly disease with a low survival rate, particularly in its advanced stages. Advanced pancreatic cancer remains a major clinical challenge due to limited treatment options. Surgical resection may not always be feasible, and traditional chemotherapy often shows restricted effectiveness. As a result, researchers are exploring a multifaceted therapeutic approach targeting the genetic and molecular drivers of the disease. A combination of molecular profiling and targeted therapies are being investigated to improve outcomes and address the shortcomings of traditional treatments. The focus of this review is to provide a summary of current and completed clinical trials for the treatment of advanced pancreatic cancer. This includes adagrasib (a KRAS inhibitor), olaparib (a PARP inhibitor for *BRCA* mutations), APG-1387 (an IAP antagonist), minnelide (an anti-stromal agent), arimastat (an MMP inhibitor), MK-0646 (an IGF1R inhibitor), sirolimus (an mTOR inhibitor), and metabolic inhibitors. These agents are being evaluated both as standalone treatments and in combination with standard therapy. Furthermore, we have summarized novel approaches such as cancer vaccines and ablation techniques as emerging strategies in the treatment of advanced pancreatic cancer. We have also examined the challenges in treating advanced pancreatic cancer and the factors contributing to therapeutic failure, which may offer valuable insights for developing more effective treatment strategies and innovative drug designs.

## Simple Summary

Pancreatic cancer is one of the deadliest forms of cancers, with limited treatment options and low survival rates. The disease is typically diagnosed at advanced stages, contributing to poor prognosis. Traditional chemotherapy often falls short, and surgery may not be a viable option for many patients with advanced pancreatic cancer. To address these challenges, new therapies targeting the genetic and molecular aspects of the disease are currently being explored. Furthermore, ongoing clinical trials are investigating a range of therapeutic approaches, including novel chemical entities, immunotherapy, and mRNA vaccines, both as standalone therapies and in combination with standard treatments, to improve patient outcomes in current and completed clinical trials for the treatment of advanced pancreatic cancer, alongside an analysis of challenges with treatment and drug failure to inform future therapeutic development.

## 1. Introduction

Pancreatic cancer remains one of the most lethal malignancies, with a 5-year relative survival rate of 12.8% [1]. In 2024, it was estimated that 66,440 new cases would be diagnosed, accounting for 3.3% of all new cancer cases with approximately 51,750 deaths, representing 8.5% of all cancer-related fatalities [1]. The low survival rate can be attributed to the inherent challenges associated with early detection and the limited efficacy of current therapeutic interventions. Many patients in their early stages of pancreatic cancer are either asymptomatic or experience nonspecific symptoms such as fatigue, nausea, vomiting, and weight loss, leading to misdiagnosis or delayed detection [2]. As a result, the disease is often diagnosed at an advanced stage when it has already progressed significantly [3], making it more challenging to treat and contributing to high mortality rates [4].

**Figure 1:**
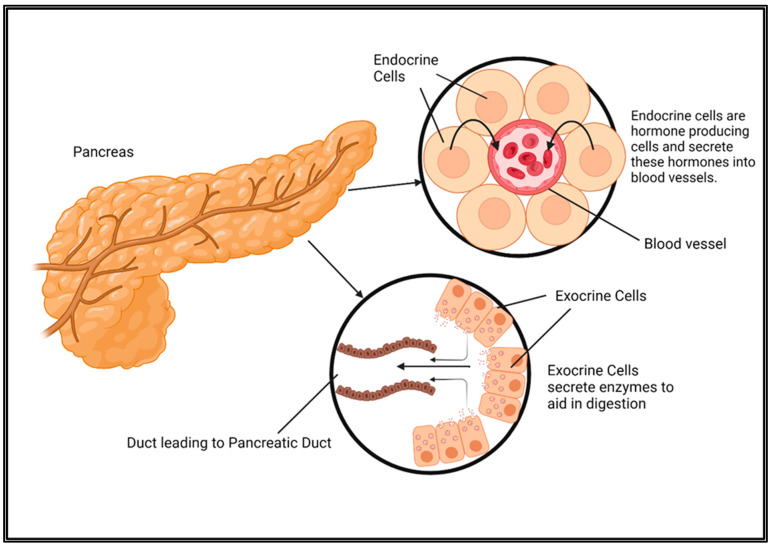
Schematic presentation of endocrine and exocrine cells [5].

Pancreatic cancer is classified into endocrine and exocrine types, each originating from distinct functional cell types within the pancreas (Figure 1). These subtypes exhibit significant differences in their biological behavior, clinical presentation, and treatment approaches.

Endocrine pancreatic cancer, also known as pancreatic neuroendocrine tumors (PNETs) or islet cell tumors, originates from the hormone-producing islets of Langerhans, which regulate metabolism, digestion, and glucose homeostasis [6]. These islets consist of five distinct cell types, each responsible for producing a specific hormone. Alpha (α) cells secrete glucagon, which raises blood glucose levels by stimulating glycogenolysis and gluconeogenesis in the liver, ensuring energy availability during fasting [6]. Beta (β) cells produce insulin, which lowers blood glucose by facilitating glucose uptake into muscle, fat, and liver cells, promoting glycogen storage, and inhibiting gluconeogenesis [6]. The dysfunction of beta cells is a key factor in the development of diabetes mellitus. Delta (δ) cells release somatostatin, a regulatory hormone that inhibits the secretion of both insulin and glucagon, thereby preventing extreme glucose fluctuations [6]. Somatostatin also suppresses gastric acid secretion and slows nutrient absorption by inhibiting other gastrointestinal hormones [6]. Epsilon (ε) cells produce ghrelin, commonly referred to as the “hunger hormone”, which stimulates appetite by acting on the hypothalamus and also influences insulin sensitivity and glucose metabolism [6]. Lastly, PP (pancreatic polypeptide) cells, formerly known as upsilon (γ) cells, secrete pancreatic polypeptide (PP), which regulates pancreatic enzyme secretion, modulates hepatic glucose metabolism, and reduces gastric motility, contributing to appetite control and digestion [6].

In contrast, exocrine pancreatic cancer, primarily pancreatic ductal adenocarcinoma (PDAC), originates from the exocrine cells of the pancreas, which produce and secrete enzymes into the small intestine to aid in digestion [7]. The pancreas releases three types of digestive enzymes via the pancreatic ducts: amylase, which breaks down carbohydrates into simpler sugars; lipase, which facilitates fat digestion by hydrolyzing triglycerides into free fatty acids and glycerol; and proteases (such as trypsin and chymotrypsin), which break down proteins into amino acids, allowing for their absorption from the small intestine [7]. These enzymes are secreted in their inactive forms, only to be activated upon reaching the intestine to prevent self-digestion of the pancreatic tissue [7]. PDAC accounts for approximately 90% of all pancreatic cancer cases and is highly aggressive, often diagnosed at advanced stages due to its asymptomatic progression [8]. The disease is primarily driven by genetic mutations that promote uncontrolled cell growth, leading to tumor formation within the pancreatic ducts [7]. As the cancer progresses, malignant cells invade surrounding tissues and metastasize to distant organs, making PDAC one of the most lethal malignancies [7]. Given this complexity, a clear understanding of the distinct functions of both endocrine and exocrine pancreatic cells is essential for identifying effective therapeutic strategies and improving patient outcomes.

To combat pancreatic cancer, Gemcitabine was initially considered the standard of care. However, about a decade ago, the treatment paradigm shifted to include nab-paclitaxel (an albumin-bound nanoparticle formulation of paclitaxel) in combination with Gemcitabine. For advanced or metastatic pancreatic cancer, first-line chemotherapy options now include FOLFIRINOX (a combination of folinic acid, fluorouracil, irinotecan, and oxaliplatin), gemcitabine/nab-paclitaxel, or NALIRIFOX (a combination of liposomal irinotecan, 5-fluorouracil/leucovorin, and oxaliplatin) regimens [4,9,10,11].

A retrospective comparative effectiveness study analyzed 1102 patients with metastatic PDAC (mPDAC) to compare FOLFIRINOX and gemcitabine plus nab-paclitaxel as first-line treatments. The cohort included 618 men (56.1%), with a median age of 60 years (IQR, 55.5–63.7 years). Patients treated with FOLFIRINOX were younger (median 59.1 vs. 61.2 years; *p* < 0.001) and had better ECOG performance status scores (39.9% vs. 32.8% with PS 0; *p* = 0.02). The median overall survival (OS) was 9.27 months (IQR, 8.74–9.76) for FOLFIRINOX and 6.87 months (IQR, 6.41–7.66) for gemcitabine plus nab-paclitaxel, demonstrating a statistically significant survival benefit for FOLFIRINOX (*p* < 0.001). This benefit was consistent across different subgroups, including age, ECOG performance status, and metastatic burden. The study concluded that FOLFIRINOX provided a survival advantage of approximately 2.4 months compared to the gemcitabine plus nab-paclitaxel regimen, along with fewer hospitalizations and lower long-term posttreatment costs, despite being associated with higher toxicity. However, the authors emphasized the need for a randomized clinical trial to further validate these findings [12].

In addition to the above treatments, targeted therapy using erlotinib in combination with gemcitabine has been established as a treatment option. Erlotinib is a tyrosine kinase inhibitor (TKI) that targets the epidermal growth factor receptor (EGFR), a receptor necessary for cancer growth and survival [4,13]. Immunotherapy has shown promise, with anti-PD-1 antibodies approved for a small subset of pancreatic cancers with specific biomarkers (MSI-H, dMMR, or TMB-high) [11]. Researchers are exploring combination strategies to increase immunogenicity and overcome the immunosuppressive tumor microenvironment. Furthermore, numerous clinical trials are currently underway to investigate novel therapeutic strategies including cancer vaccines aimed at improving survival rates and quality of life (QoL) in patients with advanced pancreatic cancer. This review provides an updated overview of these clinical trials, alongside an analysis of the factors contributing to treatment and drug failure, to better inform future therapeutic development for advanced pancreatic cancer.

## 2. Mechanisms Involved in Advanced Pancreatic Cancer Progression

**Figure 2:**
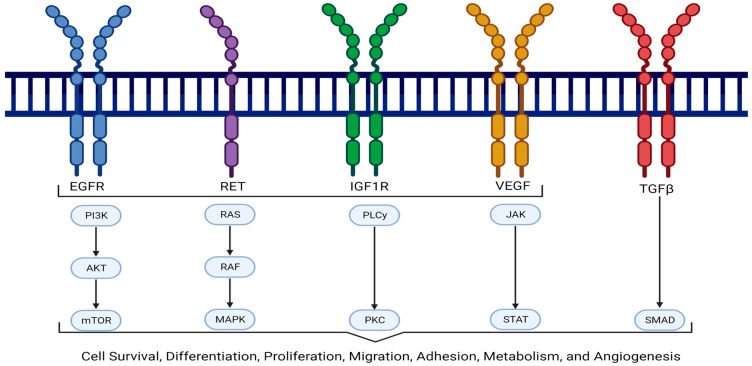
The different pathways present in advanced pancreatic cancer pathogenesis [5].

Most pancreatic cancers are characterized by an average of over 60 genetic mutations within the pancreatic tissue [14]. The most common genetic alterations in PDAC include mutations in *KRAS* (88%), *TP53* (77%), *SMAD4* (29%), *CDKN2A* (18%), and *TGFBR2* (7%) [15]. Among these, mutations in the *KRAS* gene, which controls cell growth, are the most prevalent. Particularly, point mutations in codon 12 (such as G12D or G12V) of the *KRAS* gene lead to uncontrollable cellular growth and are considered key drivers of pancreatic tumorigenesis [16]. While *KRAS* mutations are most commonly observed, some patients harbor mutations on the *BRAF* gene instead. Mutations in the *BRAF* gene or *KRAS* gene independently activate the MAPK signaling pathway, contributing to the development of pancreatic cancer [17]. Additionally, germline mutations in *BRCA* genes are associated with an increased risk of pancreatic cancer, with approximately 8% of patients with sporadic pancreatic cancer carrying these mutations [18]. Another critical pathway implicated in pancreatic tumor progression is the EGFR signaling pathway, which regulates various aspects of tumor cell behavior and contributes to resistance to chemotherapy and chemoradiation. The overexpression of EGFR on the surface of pancreatic neoplastic cells has been observed in 30–89% of patients with pancreatic cancer [19]. Some of the major signaling pathways involved in the pathogenesis of advanced pancreatic cancer are depicted in Figure 2.

Pancreatic cancer is also linked to other signaling pathways such as Wnt, TGFβ, and Notch, which are involved in the formation and maintenance of cancer stem cells. These pathways promote the survival and self-renewal of pancreatic cancer stem cells and are also believed to play a critical role in chemoresistance. The Wnt pathway regulates pancreatic stem cells, and its overactivation has been shown to inhibit apoptosis, contributing to tumor formation and progression [20]. The TGFβ pathway facilitates the epithelial–mesenchymal transition (EMT), which helps cancer cells to acquire a mesenchymal phenotype that promotes invasion and metastasis [21]. The Notch pathway controls cell growth and survival, and its overexpression influences tumor progression, stemness, and therapy resistance [22]. The overexpression of these proteins and pathways is observed in the majority of pancreatic cancer cases. As a result, several ongoing and completed clinical trials are investigating targeted therapies aimed at interrupting these signaling cascades to slow disease progression. Additional signaling pathways relevant to targeted therapy will be discussed in the following sections.

Next-generation sequencing (NGS) has revolutionized our understanding of the genetic landscape of PDAC, revealing distinct molecular subtypes and potentially actionable mutations [23]. Recent advancements in NGS techniques have allowed for the identification of two distinct molecular subtypes of PDAC: classical and basal-like, which may be predictive of responses to chemotherapy [24]. The Precision Promise platform trial is currently evaluating multiple regimens in metastatic pancreatic cancer using a phase 2/phase 3 design (NCT04229004). This innovative trial design aims to accelerate the development of new therapies by allowing multiple investigational treatments to be tested simultaneously and adapting based on ongoing results. Additionally, research is exploring the potential of using NGS on liquid biopsies to identify genetic mutations that may predict early peritoneal dissemination in PDAC patients [23].

Apart from genetic mutations, pancreatic cancer is associated with the overexpression of various proteins and enzymes. One such protein is hyaluronan (HA), a polysaccharide present in the pancreatic stroma. HA plays a key role in regulating cell proliferation, migration, and adhesion. The overexpression of HA in the tumor microenvironment contributes to cancer progression by promoting cell growth, drug resistance, and metastasis [25,26].

**Figure 3:**
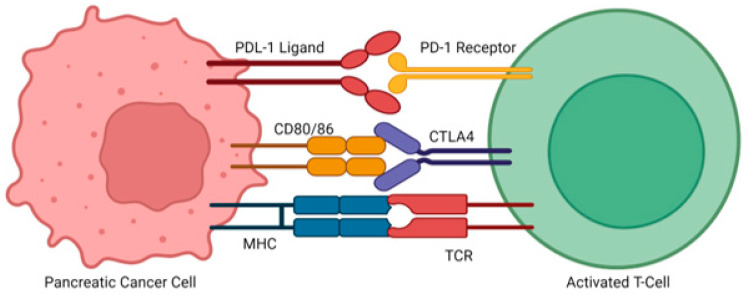
The cancer cell expresses the PDL-1 ligand, which binds to the PD-1 receptor. This eventually leads to T-cell apoptosis. CTLA4 also plays a role by inhibiting T-cell activation [5].

In addition to genetic mutations and protein overexpression, immune evasion is another critical factor that contributes to the progression of pancreatic cancer. One mechanism by which pancreatic tumors create an immunosuppressive microenvironment is through the infiltration of immunosuppressive cells such as tumor-associated macrophages, myeloid-derived suppressor cells (MDSCs), and regulatory T-cells (Tregs). These immunosuppressive cells allow the tumor to evade immune surveillance and create a tumor-permissive environment that supports cancer growth, metastasis, and resistance to immunotherapy [27]. Immune checkpoint pathways also play a key role in immune evasion. Cytotoxic T-lymphocyte–associated protein 4 (CTLA-4) binds to CD80 or CD86 on antigen-presenting cells, inhibiting T-cell activation. In additional, pancreatic tumor cells often overexpress programmed death-ligand 1 (PD-L1), which binds to PD-1 receptors on T cells. This interaction triggers T-cell apoptosis, further impairing immune-mediated tumor recognition and clearance. Tumors may also evade detection by downregulating major histocompatibility complex (MHC) molecules, which are essential for antigen presentation and T-cell recognition (Figure 3) [28,29]. Beyond immune checkpoint signaling and MHC issues, T-cell dysfunction is worsened by chronic antigen exposure in the tumor microenvironment, leading to T-cell exhaustion. Consequently, even when T cells are present, they may be unable to mount an effective anti-tumor response, allowing the tumor to persist undetected [30].

## 3. Challenges in the Treatment of Advanced Pancreatic Cancer

Detecting pancreatic cancer at an early stage remains a significant challenge due to its deep anatomical location and the absence of specific symptoms, often leading to diagnosis at an advanced stage. Routine physical examinations are ineffective in identifying early tumors, and currently, no major medical organizations recommend screening for individuals at average risk as there is no standard diagnostic tool or established early detection method that has been shown to reduce mortality [2]. However, the importance of early detection cannot be overstated, as it significantly impacts patient outcomes.

For individuals with a strong family history or a known genetic predisposition, genetic testing offers a valuable tool for assessing increased risk [2]. Those classified as high-risk individuals may undergo surveillance using endoscopic ultrasound (EUS) or magnetic resonance cholangiopancreatography (MRCP) to detect pancreatic cancer at a potentially treatable early stage [2]. When pancreatic cancer is suspected, computed tomography (CT) scans are often used to assess the pancreas and determine if the disease has spread to nearby structures or distant organs [2]. Additional diagnostic tools, such as magnetic resonance imaging (MRI), positron emission tomography (PET) scans, and tissue biopsy, can be used to confirm the diagnosis and help guide treatment planning [2].

In some cases, blood tests measuring tumor markers such as CA 19-9 and carcinoembryonic antigen (CEA) may aid in diagnosis and disease monitoring. However, these markers are not reliable for the independent detection of pancreatic cancer due to variability in their sensitivity and specificity [2]. Given the typically late-stage diagnosis and poor disease prognosis, ongoing research is focused on developing effective early-detection strategies to improve patient outcomes [2].

Low rates of successful outcomes are also observed in patients with advanced pancreatic cancer, which is most commonly diagnosed in the geriatric population [31]. Following diagnosis, treatment presents another significant challenge. Currently, chemoradiation has not demonstrated a survival benefit over chemotherapy alone [32]. Surgery remains an option in earlier stages of the disease; however, recurrence rates are extremely high, limiting its long-term effectiveness [33]. Therefore, therapies involving targeted or systemic interventions appear to be the most promising strategies for disease management. When evaluating the impact of interventional therapies, chemotherapy and other first-line therapies have shown limited effectiveness in improving survival rates, largely due to the immunosuppressive microenvironment surrounding pancreatic tumors [33]. As mentioned previously, hyaluronan is a key component of the extracellular matrix that contributes to the tumor microenvironment by promoting cell growth and facilitating immune evasion [25]. This immunosuppressive microenvironment can eventually lead to the development of chemoresistance, which is a major barrier to effective drug treatment. Another significant challenge is the heterogeneity of pancreatic cancer, which complicates the development of uniform treatment strategies [34]. Furthermore, the adverse effects associated with current therapies can significantly impact patient QoL. For example, the FOLFIRINOX chemotherapy regimen has been reported to induce neutropenia and nausea/vomiting, which may discourage both patients and clinicians from continuing or initiating treatment [35].

PDAC is notoriously resistant to drug therapy; however, recent research has identified several strategies to overcome this challenge. One approach focuses on targeting the tumor microenvironment, particularly the stiff extracellular matrix (ECM), which impairs drug delivery and promotes chemoresistance. Studies have shown that softening the ECM or inhibiting CD44 signaling can re-sensitize cancer cells to chemotherapy [36]. Another promising strategy involves targeting metabolic reprogramming in PDAC, as alterations in glucose, lipid, and amino acid metabolism contribute to therapeutic resistance. Inhibiting key enzymes or transporters involved in these pathways has shown potential in preclinical studies [37]. Additionally, the inhibition of molecular pathways such as NF-κB and multidrug resistance (MDR) proteins has shown effectiveness in reversing resistance to gemcitabine and other chemotherapeutic agents [37]. Emerging therapies also include stromal depletion through Hedgehog pathway inhibition and cancer stem cell eradication, both aimed at improving drug delivery and reducing relapse rates [37]. To further address these challenges, ongoing clinical research is focused on developing novel drugs aimed at improving survival and QoL by targeting the immunosuppressive tumor microenvironment, overcoming drug resistance, and addressing the heterogeneity that characterizes pancreatic cancer.

## 4. Ongoing and Completed Clinical Trials in the Treatment of Advanced Pancreatic Cancer

**Figure 4:**
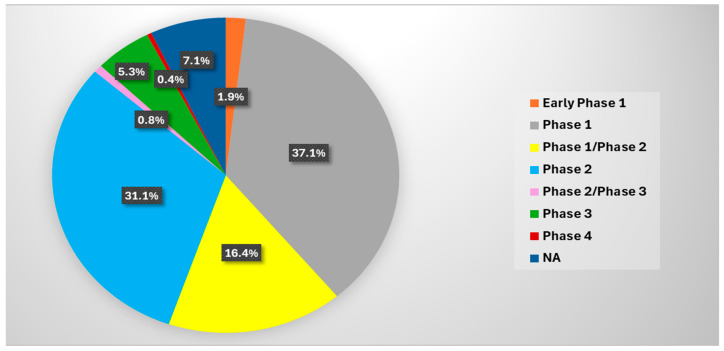
The various stages of completed and ongoing clinical trials in advanced pancreatic cancer treatment.

**Figure 5:**
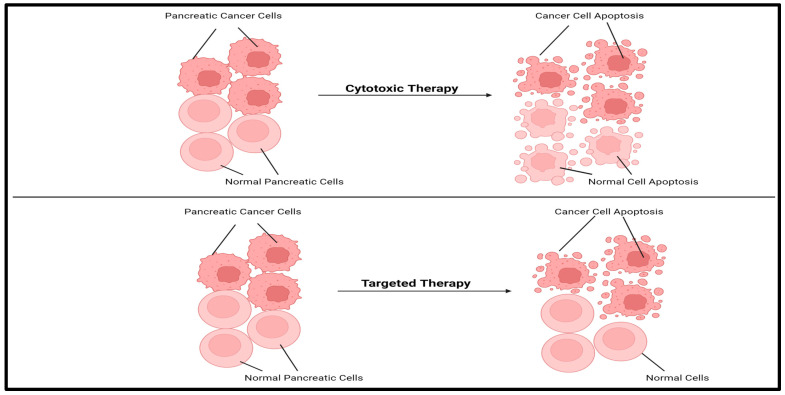
Cytotoxic vs. targeted therapies [5].

As of January 2025, a search on ClinicalTrials.gov using the keyword “Advanced Pancreatic Cancer” yielded 623 completed clinical trials, 115 active but not recruiting trials, 69 not yet recruiting trials, and 326 recruiting interventional clinical trials. These studies aim to evaluate the efficacy and safety of various drugs, drug combinations, and procedures for the treatment of advanced pancreatic cancer. Among the ongoing and completed clinical trials, 1.9% are in early phase 1, 37.1% are in phase 1, 16.4% are in phase 1/phase 2, 31.1% are in phase 2, 0.8% are in phase 2/phase 3, 5.3% are in phase 3, 0.4% are in phase 4, and 7.1% are categorized as not applicable (Figure 4). The majority of trials are in the early phases, reflecting the ongoing challenges in identifying safe and effective treatments. Nevertheless, several trials show promise. These trials are investigating cytotoxic therapies, targeted therapies, repurposed drugs, and novel procedures in an effort to determine the most effective treatment strategies. Cytotoxic therapies are non-selective and kill both cancerous and healthy cells, while targeted therapies are designed to specifically attack cancer cells (Figure 5). Repurposed drugs may fall into either category, having originally been developed for other conditions but now being tested for their potential to treat advanced pancreatic cancer.

### 4.1. Monotherapy and Combinations with Standard Therapy

Several ongoing and completed clinical trials have evaluated the use of additional drugs in combination with current standard-of-care therapies in an effort to improve their effectiveness. While standard care has provided some clinical benefits, it remains insufficient, as reflected by persistently low survival rates. The goal of combination therapy is to strengthen the efficacy of existing treatments and improve outcomes for patients with advanced pancreatic cancer. On the other hand, certain trials are investigating novel agents as standalone treatments, with the goal of establishing completely new treatment strategies.

#### 4.1.1. Cytotoxic Monotherapy or in Combination with Standard Therapy

Clinical trials investigating cytotoxic therapy have evaluated cytotoxic drugs either as monotherapy or in combination with standard-of-care treatments (Table 1). For example, a completed phase 3 trial (NCT02184195) assessed the efficacy of olaparib, a PARP inhibitor, as maintenance therapy in patients with metastatic pancreatic adenocarcinoma harboring germline *BRCA1* or *BRCA2* mutations. A total of 154 patients who had not experienced disease progression after at least 16 weeks of first-line platinum-based chemotherapy were randomized in a 3:2 ratio to receive either olaparib (300 mg twice daily; n = 92) or a placebo (n = 62) [38,39,40].

The primary endpoint, progression-free survival (PFS), was significantly improved in the olaparib group, with a median PFS of 7.4 months [IQR, 4.1–11.0], compared to 3.8 months [IQR, 3.5–4.9] in the placebo group. This substantial improvement, reflected by a hazard ratio (HR) of 0.53 (95% CI, 0.35–0.82; *p* = 0.004), underscores the potential of olaparib to effectively delay disease progression in this patient population.

Regarding safety, the median duration of treatment was 6.0 months (a range of 0.8–45.3) in the olaparib group and 3.7 months (a range of 0.1–30.1) in the placebo group. Serious adverse events (SAEs) occurred in 24% of olaparib-treated patients compared to 15% in the placebo group; however, the overall safety profile was considered acceptable. Treatment discontinuation due to adverse events occurred in 5% of patients receiving olaparib and 2% of those receiving placebo. Notably, no cases of myelodysplastic syndrome (MDS) or acute myeloid leukemia (AML) were reported in either group.

Patient-reported outcomes indicated no clinically meaningful differences in health-related QoL (HRQoL) between the treatment arms. Based on these findings, olaparib was approved as a first-line maintenance therapy for patients with germline BRCA-mutated metastatic pancreatic cancer [38,39,40].

Before the introduction of nab-paclitaxel in combination with gemcitabine, gemcitabine monotherapy was considered the standard care of treatment. Hence, many clinical trials focused on identifying agents that could be combined with gemcitabine to improve clinical outcomes. Drugs such as Etoposide and CI-994 were investigated prior to the approval of nab-paclitaxel as a first-line treatment alongside gemcitabine (NCT00202800, NCT00004861). Notably, before nab-paclitaxel received approval, cisplatin demonstrated promising results when combined with gemcitabine, showing improvements in both progression-free survival and overall survival compared to gemcitabine monotherapy [41]. A phase 2 randomized trial evaluated the efficacy and safety of gemcitabine plus cisplatin (GemCis) vs. gemcitabine alone (Gem) in patients with advanced pancreatic cancer. A total of 195 patients were enrolled, with 97 patients receiving GemCis and 98 patients receiving Gem. Baseline characteristics were well balanced between the two treatment groups [41].

The study demonstrated a promising trend in PFS, with the GemCis group (5.3 months) showing a median PFS of 5.3 months compared to 3.1 months in the Gem group, yielding a hazard ratio (HR) of 0.75 (*p* = 0.053), indicating a trend toward statistical significance [41]. The median overall survival (OS) was also higher in the GemCis group (7.5 months vs. 6.0 months), although the difference did not reach statistical significance (HR = 0.80; *p* = 0.15) [41].

Tumor response rates were comparable between the two groups (10.2% for GemCis vs. 8.2% for Gem), indicating that both treatments are effective. However, the rate of stable disease was significantly higher in the combination group (60.2% vs. 40.2%; *p* < 0.001), suggesting improved disease control with the dual therapy. Grade 3 to 4 hematologic toxicity remained below 15% in both arms, indicating that the combination regimen was generally well-tolerated [41]. As a result, cisplatin combined with gemcitabine is now considered one of the preferred treatment regimens for metastatic pancreatic cancer [4].

A comprehensive meta-analysis of 15 studies, including 13 in the quantitative synthesis, evaluated the efficacy and safety of combining gemcitabine with capecitabine (GemCap) vs. gemcitabine monotherapy (Gem) in patients with advanced pancreatic cancer [42,43]. The pooled hazard ratio (HR) for overall survival (OS) favored the GemCap regimen, with an HR of 0.85 (95% confidence interval [CI]: 0.75–0.95; *p* = 0.007), indicating a 15% reduction in the risk of death compared to Gem alone [42]. PFS was also improved in the combination therapy group, with a pooled HR of 0.80 (95% CI: 0.72–1.04; *p* = 0.0002) [42]. The one-year survival rate for patients receiving GemCap was 33.1% (95% CI: 28.7–37.5), and the objective response rate (ORR) was 22.9% (95% CI: 17.6–28.3). The disease control rate (DCR) was notably higher in the GemCap group at 65.7% (95% CI: 56.7–74.8). When compared to Gem monotherapy, the combination therapy demonstrated significantly higher ORR (odds ratio [OR]: 1.98; 95% CI: 1.34–2.67; *p* = 0.0003) and DCR (OR: 1.41; 95% CI: 1.05–1.88; *p* = 0.02) [42]. These findings suggest that GemCap may offer a clinically meaningful survival and response advantage in the treatment of advanced pancreatic cancer.

These findings have important implications for clinical practice, suggesting that the GemCap combination may be a more effective treatment option for patients with advanced pancreatic cancer. In terms of safety, the most common grade ≥ 3 hematological toxicities observed in the GemCap group were neutropenia (19.7%), leukocytopenia (7.9%), and anemia (4.9%). Non-hematological toxicities of grade ≥ 3 included hand–foot syndrome (6.3%), fatigue (5.7%), and nausea (4.8%) [42]. These results suggest that while the GemCap combination offers improved efficacy over gemcitabine alone, it is associated with a higher incidence of certain adverse events. However, with appropriate supportive care and medical intervention, these adverse events can be effectively managed, and the survival benefits of the GemCap regimen may outweigh the associated risks. As a result, capecitabine in combination with gemcitabine is now considered one of the recommended treatment regimens for metastatic pancreatic cancer [4].

Currently, many completed and ongoing clinical trials are investigating the effects of various cytotoxic drugs when combined with the standard therapy of gemcitabine and nab-paclitaxel. One such ongoing phase 1/phase 2 clinical trial (NCT04643405) is evaluating the safety and efficacy of APG-1387 in combination with gemcitabine and nab-paclitaxel. APG-1387 is a small-molecule compound that targets inhibitors of apoptosis proteins (IAPs) to promote apoptosis in cancer cells. This study appears promising, as APG-1387 has already demonstrated potent antitumor activity in nasopharyngeal cancer [45]. Another active phase 1 clinical trial (NCT04046887) [44] is examining the safety and toxicity of adding Lonsurf (a fixed-dose combination medication of trifluridine and tipiracil) to standard Gemcitabine and nab-paclitaxel Lonsurf has previously been shown to extend PFS by 1.8 months in patients with metastatic colorectal cancer [46]. However, a prior terminated phase 2 trial (NCT02921737) evaluating Lonsurf as monotherapy in advanced pancreatic cancer yielded no significant benefit, as all participants died before the study’s completion. The limited number of participants in that study raises the possibility that a larger trial could yield different results. Despite these mixed outcomes, there is still significant potential for further research into cytotoxic agents that could improve the effectiveness of standard therapy, especially considering the relatively limited number of clinical trials dedicated to this therapeutic approach.

#### 4.1.2. Targeted Therapy as a Monotherapy or in Addition to Standard Therapy

**Figure 6:**
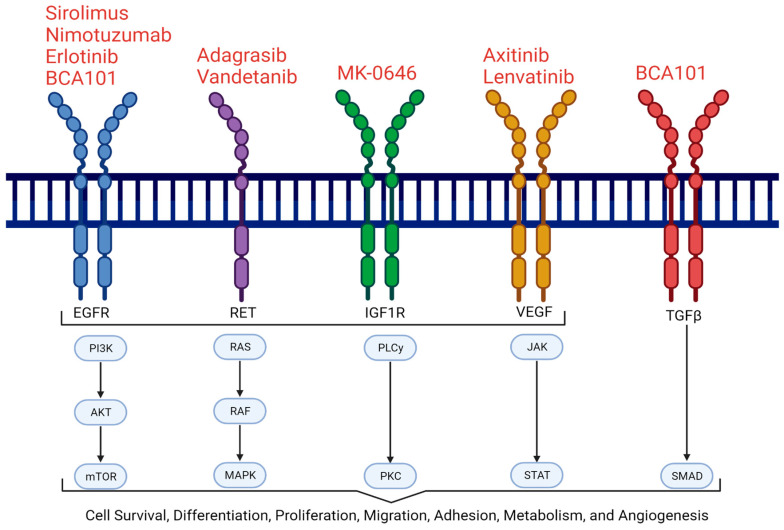
Targeted therapies for different pathways involved in the pathogenesis of advanced pancreatic cancer [5].

Targeted therapies are being explored as monotherapy and in combination with standard treatments for advanced pancreatic cancer (Figure 6) (Table 2). As discussed previously, among the most promising molecular targets, *KRAS* mutations are highly prevalent in PDAC, occurring in approximately 85% of cases [47]. Several KRAS inhibitors are currently undergoing clinical trials across multiple cancer types, including pancreatic cancer [48]. The KRAS protein cycles between an inactive GDP-bound form and an active GTP-bound form. Mutations in codon 12 result in constitutive activation of *KRAS*, driving uncontrolled cell proliferation [49]. KRAS inhibitors can be broadly categorized into KRAS-OFF and KRAS-ON inhibitors.

KRAS-OFF inhibitors, such as sotorasib and adagrasib, covalently bind to the cysteine residue at KRAS G12C, locking the protein in its inactive GDP-bound form [49]. These agents have shown clinical efficacy in non-small cell lung cancer but are limited to cancers harboring the G12C mutation, which accounts for approximately 13% of lung adenocarcinomas [49]. In the KRYSTAL-1 trial (NCT03785249), adagrasib demonstrated encouraging clinical activity in previously treated patients with PDAC harboring *KRAS* G12C mutations. The objective response rate was 35.1%, with a median PFS of 7.4 months and a median overall survival (OS) of 14.0 months [50]. Additionally, a phase 1b trial (NCT05634525) is currently underway to evaluate adagrasib as a monotherapy in metastatic pancreatic cancer. The trial focuses on assessing safety and preliminary efficacy in patients with *KRAS* G12C mutations. Similarly, the CodeBreaK 100 trial (NCT03600883) showed that sotorasib exhibited clinically meaningful antitumor activity in heavily pretreated patients with advanced *KRAS* G12C-mutant PDAC [51]. The CodeBreaK 101 trial (NCT04185883) is currently recruiting and aims to evaluate sotorasib in combination with other agents in patients with advanced solid tumors, including PDAC harboring NCT00769483 G12C mutations.

Adagrasib and sotorasib are approved for the treatment of non-small cell lung cancer. It will be interesting to see how these drugs perform against advanced pancreatic cancer (NCT05634525 and NCT04185883) [66].

KRAS-ON inhibitors target the active, GTP-bound state of KRAS. One such agent, MRTX1133, is a non-covalent KRAS G12D inhibitor capable of suppressing the oncoprotein in both its active (ON) and inactive (OFF) states. It has demonstrated potent anti-tumor activity in preclinical studies, particularly in PDAC [16]. Another promising KRAS-ON inhibitor, HRS-4642, also targets the active GTP-bound form of KRAS G12D and is currently progressing through clinical development for advanced pancreatic cancer.

A phase 1/phase 2 trial (NCT06427239) is actively recruiting patients to evaluate the combination of HRS-4642 with adebelimab, a PD-L1 inhibitor, in unresectable or metastatic PDAC. Additional upcoming trials are planned to explore novel HRS-4642-based combinations, although recruitment has not yet begun. These include a Phase 2 trial (NCT06547736) combining HRS-4642 with an antibody–drug conjugate (ADC) and two phase 1/phase 2 trials pairing HRS-4642 with nimotuzumab, an EGFR inhibitor (NCT06773130) or in combination with chemotherapy (NCT06770452). Collectively, these studies represent a strategic shift toward combination regimens aimed at overcoming KRAS-driven resistance mechanisms in PDAC. The results will be critical in determining HRS-4642’s potential to enhance treatment outcomes in advanced pancreatic cancer.

Pan-RAS inhibitors—also referred to as tri-complex inhibitors or RAS(ON) multi-selective inhibitors—such as RMC-7977 and RMC-6236, have emerged as promising therapeutic agents for RAS-driven cancers. These compounds exhibit broad activity against both mutant and wild-type forms of *KRAS*, *NRAS*, and *HRAS* in their GTP-bound state [67,68]. The tri-complex mechanism involves linking cyclophilin A to active RAS, thereby blocking RAS–effector interactions and suppressing downstream signaling [69]. Among these, the pan-RAS inhibitor daraxonrasib (RMC-6236) has shown encouraging early activity and a manageable safety profile in patients with *RAS*-mutant PDAC. In an ongoing phase 1/phase 1b trial (NCT05379985), RMC-6236 has produced objective responses in patients with advanced *KRAS*-mutant lung and pancreatic adenocarcinomas [69].

While KRAS G12C inhibitors such as sotorasib and adagrasib have shown modest improvements in survival for the small subset of PDAC patients with G12C mutations, newer G12D-targeting agents may offer broader therapeutic benefits. For example, QTX3034 is a highly selective, non-covalent, orally bioavailable multi-KRAS inhibitor with strong activity across several KRAS variants, particularly G12D. It is currently being evaluated in a phase 1 trial for patients with advanced solid tumors harboring *KRAS* G12D mutations (NCT06227377) [55].

Resistance to KRAS inhibitors remains a major challenge, often resulting from various mechanisms, including tumor heterogeneity and the survival of subpopulations of cells that persist despite KRAS inhibition [70,71]. Recent research indicates that resistance may be driven by the persistence of “classical-state” tumor cells, which are less responsive to KRAS-targeted therapies [72]. Preclinical studies suggest that combining KRAS inhibitors with chemotherapy or other targeted agents such as CDK4/6 inhibitors may enhance therapeutic efficacy by simultaneously targeting both basal and classical tumor cell populations [71,72,73].

Ongoing clinical trials are investigating combination strategies to overcome resistance mechanisms, including the use of SOS1 inhibitors (e.g., BI 1701963) and SHP2 inhibitors (e.g., RMC-4630). These agents aim to disrupt upstream signaling pathways involved in KRAS activation and may help restore sensitivity to KRAS inhibition (NCT04973163) [56,74].

The combination of KRAS G12C and SOS1 inhibition has shown promise in prolonging anti-tumor responses in KRAS G12C-driven cancers [75]. These ongoing studies and combination strategies aim to overcome resistance mechanisms and improve outcomes for patients with *KRAS*-mutant pancreatic cancer, potentially transforming the treatment landscape for this challenging disease.

In parallel, HA plays a crucial role in pancreatic cancer progression by contributing to the desmoplastic reaction and hindering effective drug delivery [25]. Preclinical studies in mouse models showed that Minnelide, a triptolide prodrug that interferes with HA synthesis, decreased tumor burden more effectively than gemcitabine [76]. A phase 2 trial evaluated the effects of Minnelide in patients with advanced pancreatic cancer (NCT03117920); however, no study results have been posted for this study. PEGPH20, a PEGylated human hyaluronidase, targets HA, a key component of the extracellular matrix that contributes to tumor progression and drug resistance in pancreatic cancer [77]. By enzymatically depleting hyaluronan, PEGPH20 (PEGylated recombinant human hyaluronidase) enhances the intratumoral delivery of therapeutic agents. It is currently being evaluated in combination with gemcitabine and nab-paclitaxel in a phase 2 clinical trial (NCT02921022). While preclinical studies demonstrated improved tumor perfusion and therapeutic efficacy, results from earlier clinical trials, such as the HALO 301 study (phase 3 trial, NCT02715804) [65]), showed limited benefit in overall survival despite modest improvements in progression-free survival among patients with HA-high tumors. These findings highlight the potential of PEGPH20 in select patient subsets while also underscoring the need for further investigation into its clinical utility.

Another approach targeting the tumor microenvironment involves marimastat, a matrix metalloproteinase (MMP) inhibitor. In a phase 2 trial, marimastat demonstrated safety and tolerability in patients with advanced pancreatic cancer. Although it did not significantly improve overall survival compared to gemcitabine, there was evidence of a dose–response relationship [53,54].

The mTOR signaling pathway also promotes cancer cell growth in pancreatic cancer [78]. A phase 2 trial is currently underway to assess sirolimus, a selective mTOR inhibitor, as a monotherapy for the treatment of advanced pancreatic cancer (NCT03662412). Sirolimus has shown promise in treating lung cancer and could be a viable option for advanced pancreatic cancer if the results are positive [79].

Building on the understanding of mTOR signaling in pancreatic cancer, researchers have also explored the role of insulin-like growth factor 1 receptor (IGF-1R) in disease progression. IGF-1R plays a critical role in promoting pancreatic cancer cell proliferation, survival, and drug resistance by activating key downstream signaling pathways, including PI3K/AKT/mTOR and MEK/ERK [80]. Preclinical research has demonstrated that silencing IGF-1R inhibits pancreatic cancer growth and metastasis by suppressing proliferation, invasion, migration, and colony formation, while also inducing apoptosis [60]. However, clinical trials of IGF-1R inhibitors in pancreatic cancer have produced mixed results. A phase 1/phase 2 study evaluated MK-0646, an IGF-1R monoclonal antibody, in combination with gemcitabine with or without erlotinib in patients with metastatic pancreatic cancer (NCT00769483) [60,61]. The study found that the combination of gemcitabine and MK-0646 improved overall survival compared to the standard regimen of gemcitabine plus erlotinib, although progression-free survival was reduced. Recent research has also explored the potential of combining IGF-1R inhibition with other targeted therapies, such as ERK inhibitors, to enhance therapeutic efficacy in pancreatic cancer [81].

EGFR inhibitors have also been extensively studied in the treatment of pancreatic cancer. EGFR is overexpressed in a major subset of pancreatic tumors and contributes to tumor proliferation, metastasis, and angiogenesis [82]. The addition of erlotinib, an EGFR tyrosine kinase inhibitor, to gemcitabine demonstrated a small but statistically significant survival benefit in patients with advanced pancreatic cancer [83]. This finding prompted further investigation into EGFR-targeted therapies, including monoclonal antibodies such as nimotuzumab. It has been approved to treat adult and pediatric gliomas, nasopharyngeal carcinoma, and esophageal cancer; thus, it is quite promising in the realm of pancreatic cancer [57,84]. A completed phase 2/phase 3 study (NCT00561990) evaluated the combination of nimotuzumab with gemcitabine vs. gemcitabine plus placebo in patients with unresectable metastatic or locally advanced pancreatic cancer (LAPC). The trial demonstrated improved one-year overall survival (34% vs. 19%) and progression-free survival (22% vs. 10%) for the nimotuzumab-gemcitabine group compared to gemcitabine plus placebo. Notably, patients with *KRAS* wild-type tumors experienced significantly better overall survival than those with *KRAS* mutations (11.6 vs. 5.6 months, *p* = 0.03 [58]. Another completed phase 3 NOTABLE trial (NCT02395016) demonstrated that the addition of nimotuzumab to gemcitabine significantly improved overall survival in patients with *KRAS* wild-type advanced pancreatic cancer [85]. Building on this, several new trials are underway or planned, investigating innovative drug combinations for pancreatic cancer. These include (a) NCT06429904 (active, not recruiting), a phase 2 trial evaluating the clinical efficacy and safety of nimotuzumab in combination with NALIRIFOX for the treatment of LAPC; (b) the previously mentioned NCT06770452 (not yet recruiting), a phase 2 trial investigating the effectiveness of HRS-4642 in combination with nimotuzumab and gemcitabine + nab-paclitaxel as a first-line treatment for advanced pancreatic cancer with *KRAS* G12D mutation; and (c) NCT06404840 (recruiting), a phase 2 trial assessing nimotuzumab combined with gemcitabine + nab-paclitaxel in patients with pancreatic cancer and liver metastases. These trials represent cutting-edge efforts to improve treatment outcomes in this challenging malignancy.

Following the discussion of EGFR’s role in pancreatic cancer, emerging research highlights the critical interplay between HER2 and HER3 in driving tumor progression and therapeutic resistance [86]. In this regard, zenocutuzumab (MCLA-128) (Bizengri), a bispecific antibody targeting HER2 and HER3, has shown promising results in treating *NRG1* fusion-positive cancers, particularly non-small cell lung cancer (NSCLC) and pancreatic adenocarcinoma. The FDA granted accelerated approval to Bizengri on 4 December 2024 for adults with advanced, unresectable, or metastatic NSCLC or pancreatic adenocarcinoma harboring *NRG1* gene fusions [87]. This approval was based on the phase I/II eNRGy trial (NCT02912949), which demonstrated durable antitumor activity across multiple tumor types. In the eNRGy trial, the overall response rate was 42% for pancreatic adenocarcinoma and 29% for NSCLC, with a median duration of response of 11.1 months [59].

Furthermore, the *RET* (rearranged during transfection) proto-oncogene has been investigated as a potential therapeutic target in pancreatic cancer. *RET* encodes a receptor tyrosine kinase involved in regulating cell survival, proliferation, and migration. Although *RET* fusions are rare in pancreatic cancer, occurring in approximately 0.6–1.9% of cases, wild-type RET and its co-receptors are overexpressed in a significant subset of tumors. This overexpression contributes to tumorigenesis and perineural invasion through activation by ligands such as GDNF and Artemin [88,89]. The Vandetanib in Pancreatic Cancer (ViP) trial (NCT02395016), a phase 2 double-blind, randomized study, evaluated vandetanib, a multi-kinase inhibitor targeting RET, VEGFR2, and EGFR in combination with gemcitabine vs. gemcitabine plus placebo in patients with advanced pancreatic cancer. The trial found no significant improvement in overall survival with vandetanib (8.83 months vs. 8.95 months for placebo; HR = 1.21; *p* = 0.303) [62]. Despite these results, tyrosine kinase inhibitors like vandetanib remain of interest due to their potential to target specific subsets of pancreatic cancer patients with aberrant RET signaling [89,90]. Ongoing research aims to identify predictive biomarkers and refine patient selection to maximize the therapeutic benefit of RET-targeted therapies.

Axitinib (AG-013736), a VEGFR inhibitor, was also evaluated in combination with gemcitabine vs. gemcitabine monotherapy. Two separate phase 2 and 3 trials (NCT00471146 and NCT00219557) found that the addition of axitinib to gemcitabine did not improve survival compared to gemcitabine alone. These results have contributed to the view that therapies targeting the VEGFR pathway may be ineffective in the treatment of advanced pancreatic cancer [63,64,91].

#### 4.1.3. Metabolic Inhibitors as a Monotherapy or in Addition to Standard Therapy

In addition to targeting various ligands, receptors, and matrix proteins involved in pancreatic cancer pathogenesis, metabolic inhibitors are also being actively investigated as a strategy to exploit vulnerabilities in tumor metabolism (Table 3) [92,93]. Pancreatic cancer cells undergo significant metabolic reprogramming to adapt to the nutrient-deprived and hypoxic conditions within the tumor microenvironment [94]. This adaptation involves alterations in glucose, amino acid, and lipid metabolism pathways, enabling cancer cells to sustain growth under harsh conditions [95] One key metabolic alteration in pancreatic cancer is the rewiring of glutamine metabolism, which has emerged as a promising therapeutic target [96,97,98]. Telaglenastat hydrochloride (CB-839 HCl), a glutaminase inhibitor, demonstrated safety and pharmacodynamic target engagement in a phase 1 trial, achieving disease control in 43% of patients (NCT02071862) [99]. A phase 2 basket trial (NCT03872427, BeGIN Study) is currently evaluating its efficacy in tumors harboring *NF1*, *KEAP1/NRF2*, or *STK11/LKB1* mutations, based on preclinical evidence that *KRAS*-driven pancreatic cancers with *KEAP1/NRF2* alterations rely heavily on glutaminolysis [98].

Researchers have also focused on other key metabolic enzymes in pancreatic cancer. Pyruvate dehydrogenase (PDH) and α-ketoglutarate dehydrogenase (α-KGDH) are critical components of the tricarboxylic acid (TCA) cycle and are often dysregulated in advanced pancreatic cancer, contributing to metabolic reprogramming and tumor progression [103,104]. Devimistat (CPI-613), a mitochondrial metabolism inhibitor targeting both PDH and α-KGDH, has shown synergistic activity with radiation in preclinical models [105]. As a single agent, devimistat has been evaluated in multiple phase 1 studies, including in combination with modified FOLFIRINOX (mFOLFIRINOX) for pancreatic cancer (NCT01835041) [100]. Based on encouraging results from early-phase studies, further investigation led to the phase 3 AVENGER 500 trial (NCT03504423) [100], which evaluated the efficacy of devimistat plus mFOLFIRINOX vs. standard FOLFIRINOX in patients with metastatic pancreatic cancer. However, the trial did not meet its primary endpoint of improved overall survival. Median overall survival was 11.10 months for the devimistat group vs. 11.73 months for the standard FOLFIRINOX group (HR = 0.95; *p* = 0.655). While the results have been modest, research into devimistat remains ongoing, with continued interest in optimizing its use. Current clinical trials include its combination with chemoradiation (NCT05325281) and hydroxychloroquine plus chemotherapy (NCT05733000). However, a separate trial evaluating devimistat with mFOLFIRINOX (NCT05926206) was withdrawn. These ongoing studies aim to further investigate devimistat’s potential in combination regimens and alternate dosing strategies to improve treatment outcomes in pancreatic cancer.

Methionine adenosyltransferase 2A (MAT2A) plays a critical role in advanced pancreatic cancer by promoting tumor growth and survival through the increased production of S-adenosylmethionine (SAM), the universal methyl donor essential for numerous metabolic processes [106]. In pancreatic cancer, MAT2A expression is upregulated, contributing to cancer cell proliferation and resistance to therapy [106]. AG-270, an oral MAT2A inhibitor, has been shown to reduce tumor growth in preclinical pancreatic cancer models [102]. It also demonstrated target engagement, evidenced by reduced SAM level, in a phase 1 clinical trial involving pancreatic cancer patients with MTAP deletions (NCT03435250).

SM-88 (racemetyrosine) is a novel metabolism-based therapy designed to exploit the Warburg effect by disrupting tyrosine-mediated metabolic pathways in cancer cells. It interferes with the synthesis of key proteins, such as Mucin 1 (MUC1), leading to oxidative stress, decreased anti-apoptotic signaling, and enhanced immune recognition of tumor cells. The TYME-88-Panc trial (NCT03512756) was a randomized, open-label phase 2/phase 3 study evaluating the efficacy and safety of SM-88 in combination with MPS (methoxsalen, phenytoin, and sirolimus) in patients with advanced pancreatic cancer who had received at least two prior lines of systemic therapy. While the phase 2 trial showed promising survival outcomes and favorable tolerability in heavily pretreated patients, exploratory analysis revealed that passively collected biometric data, such as step counts, correlated with self-reported QoL during the first two weeks of treatment, suggesting potential utility for wearable devices in monitoring patient well-being. Despite these encouraging findings, the trial was ultimately terminated by the sponsor. However, the Precision Promise platform trial (NCT04229004), which is currently ongoing, uses adaptive randomization to efficiently evaluate multiple treatment regimens, including the metabolic inhibitor SM-88, in patients with metastatic pancreatic cancer [107]

These efforts reflect a growing focus on disrupting metabolic dependencies while addressing challenges such as chemoresistance associated with metabolic reprogramming [108].

#### 4.1.4. Immunotherapy Treatments as a Monotherapy or in Addition to Standard Therapy

Immunotherapy is a treatment that enhances the body’s immune response to target and eliminate cancer cells, rather than attacking the cancer directly like traditional therapies [109]. This approach to treatment is gaining traction as numerous trials have recently begun evaluating immunotherapy drugs as potential treatment options for advanced pancreatic cancer (Table 4). Some clinical trials are focused on pembrolizumab, a drug that blocks the PD-1 pathway to enhance the immune system’s ability to recognize and attack cancer cells [110]. By blocking the PD-1 pathway, pembrolizumab reduces the ability of cancer cells to evade detection by T cells. It has been tested in combination with several investigational agents in clinical trials, including XL888, an Hsp90 inhibitor that may disrupt proteins required for tumor growth (NCT03095781); BCA101, a bifunctional agent targeting EGFR and TGF-β (NCT04429542); NGM831, an ILT3 antagonist antibody designed to modulate myeloid cell activity (NCT05215574); Lenvatinib, a VEGFR inhibitor with anti-angiogenic properties (NCT05273554); SD-101, a TLR9 agonist that stimulates innate immune responses (NCT05607953); NT-17, a therapy promoting T-cell development (NCT04332653); and ONC-392, a humanized anti-CTLA-4 IgG1 monoclonal antibody (NCT04140526) [111,112,113,114,115,116]. When combined with these agents, pembrolizumab has the potential to serve as a more effective immunotherapeutic strategy by targeting multiple aspects of tumor growth and immune evasion. Similarly, durvalumab is an immune checkpoint inhibitor that blocks the PD-L1 pathway, preventing it from binding to PD-1 and thereby restoring T-cell activity against tumor cells [117]. Durvalumab is also currently being evaluated in clinical trials, including in combination with tazemetostat, an EZH2 inhibitor (NCT04705818). Another trial (NCT05187338) involves the combination of durvalumab (a PD-L1 inhibitor), pembrolizumab (a PD-1 inhibitor), and ipilimumab (a CTLA-4 inhibitor). This triple immunotherapy approach may offer a promising strategy to enhance antitumor immune responses by targeting multiple immune checkpoints simultaneously. In addition, CAR-T cell therapy, which involves engineering a patient’s T cells to express chimeric antigen receptors that recognize and bind to tumor-associated antigens, has also been explored in the treatment of pancreatic cancer [118]. Several types of CAR-T cells are currently being evaluated in clinical trials, including U87 CAR-T cells (NCT05605197), IM92 CAR-T cells (NCT05275062), and IM96 CAR-T cells (NCT05287165). These trials aim to identify the most effective CAR-T constructs for treating pancreatic cancer. Immunotherapy continues to evolve and holds significant promise for the development of innovative treatment strategies for advanced pancreatic cancer.

PT886 (Spevatamig) is a novel bispecific antibody targeting Claudin 18.2 (CLDN18.2) and CD47, representing a promising approach in targeted therapy for pancreatic cancer. CLDN18.2 is a tight junction protein overexpressed in several malignancies, including pancreatic adenocarcinoma, while CD47 functions as a “do not eat me” signal that inhibits macrophage-mediated phagocytosis of cancer cells. By simultaneously targeting these two pathways, PT886 aims to enhance tumor cell elimination through multiple mechanisms, including antibody-dependent cellular cytotoxicity (ADCC) and phagocytosis [119].

The TWINPEAK study (NCT05482893) is an ongoing phase 1/phase 2 multicenter clinical trial evaluating PT886 in patients with advanced gastric, gastroesophageal junction, and pancreatic adenocarcinomas. The study is designed to assess the safety, tolerability, pharmacokinetics, and preliminary efficacy of PT886 as monotherapy and in combination with chemotherapy and/or pembrolizumab. The trial began in 2023 and is expected to complete by April 2026. The potential of PT886 in pancreatic cancer treatment is further supported by its Fast Track designation from the FDA for metastatic CLDN18.2-positive pancreatic adenocarcinoma [120].

Building on advances in immunotherapy for pancreatic cancer, cancer vaccines have emerged as a promising approach to stimulate the immune system against this aggressive disease. Unlike traditional vaccines that prevent infections, cancer vaccines are designed to treat existing cancers or prevent their recurrence. In pancreatic cancer, these vaccines work by delivering tumor antigens in various forms, such as autologous tumor cells (whole-cell vaccines), tumor-associated proteins or peptides (peptide-based vaccines), dendritic cells as delivery vectors, or mRNA encoding tumor-specific antigens (mRNA vaccines). Once introduced into the body, these antigens are processed by dendritic cells and presented to T cells, activating antigen-specific cytotoxic T cells capable of recognizing and eliminating pancreatic cancer cells [121,122].

GVAX is a cell-based vaccine composed of whole tumor cells genetically modified to secrete granulocyte-macrophage colony-stimulating factor (GM-CSF), which is intended to stimulate anti-tumor immune responses. Previous studies have shown that GVAX can induce intratumoral tertiary lymphoid aggregates, which are associated with improved survival in pancreatic cancer patients [123]. However, a phase 2 trial (NCT01896869) combining GVAX with ipilimumab as maintenance therapy did not improve overall survival compared to continued chemotherapy in metastatic pancreatic cancer [124].

Another phase 2 clinical trial (NCT02648282) evaluated the combination of cyclophosphamide, pembrolizumab, the GVAX pancreas vaccine, and stereotactic body radiation therapy (SBRT) in patients with LAPC. This study demonstrated evidence of immune activation and partial responses in some patients.

The GV1001 vaccine, a telomerase peptide vaccine, was evaluated in the TeloVac trial, a phase 3 study involving 1062 patients with advanced pancreatic cancer. Unfortunately, the addition of GV1001 did not improve overall survival compared to chemotherapy alone (NCT00425360). However, other peptide-based vaccines have shown promise. A phase 1 trial of a personalized peptide neoantigen vaccine (iNeo-Vac-P01) in seven patients with advanced pancreatic cancer demonstrated both safety and potential efficacy, with a mean overall survival of 24.1 months (NCT03645148) [125]. Beyond peptide-based vaccines, autogene cevumeran, a personalized mRNA vaccine, has shown encouraging results in early-phase trials. A phase 1 study evaluated this vaccine in 16 patients with surgically resected PDAC. The results showed that autogene cevumeran induced strong neoantigen-specific T-cell responses in 50% of participants, with some responses persisting for up to three years post-treatment [126]. Building on these findings, a phase 2 clinical trial (NCT05968326) is currently evaluating autogene cevumeran in combination with checkpoint inhibitors and chemotherapy in patients with resectable PDAC. Initiated in July 2023, the trial aims to enroll approximately 260 patients globally.

#### 4.1.5. Repurposed Drugs to Treat Advanced Pancreatic Cancer

Some drugs originally developed for other diseases are being repurposed for the treatment of pancreatic cancer (Table 5). This approach is gaining increasing recognition due to the substantially reduced regulatory hurdles associated with FDA approval for repurposed drugs compared to those required for new drug development. A phase 1 recruiting trial (NCT03889795) is evaluating the repurposing of metformin, digoxin, and simvastatin for the treatment of advanced pancreatic cancer. Traditionally, metformin is used to treat type 2 diabetes, digoxin for heart arrhythmias, and simvastatin for high cholesterol. This drug combination has shown preclinical efficacy by effectively targeting Pancreatic and Duodenal Homeobox 1 (PDX1), an oncogenic transcription factor and Baculoviral IAP Repeat-Containing 5 (BIRC5), also known as survivin, an inhibitor of apoptosis. In mouse models bearing human pancreatic tumors derived from the PANC-1, MiaPaCa-2, and Capan-2 cell lines, this combination reduced tumor growth without causing observable toxicity. By simultaneously targeting oncogenic transcription and apoptotic resistance pathways, this repurposed drug regimen holds promise as a novel therapeutic strategy for advanced pancreatic cancer [127]. Similarly, auranofin, an anti-rheumatic agent, has shown potential for repurposing in the treatment of advanced pancreatic cancer. In preclinical studies using mouse models, auranofin was found to inhibit tumor growth. These promising results suggest that auranofin may advance to clinical trials in the near future [128]. Another repurposed drug is parbendazole, an anthelmintic agent traditionally used to treat intestinal parasites. In vitro studies have shown that parbendazole induces apoptosis in pancreatic cancer cells, which may contribute to reduced tumor progression in advanced pancreatic cancer [129,130]. These findings suggest that parbendazole could be further evaluated in vivo to assess its efficacy and safety in treating advanced pancreatic cancer. Drug repurposing may offer a promising strategy to help overcome the challenges associated with this aggressive disease.

### 4.2. Emerging Approaches in Pancreatic Cancer Management

Pancreatic cancer treatment has evolved significantly in recent years, with novel delivery methods, advanced procedures, and innovative clinical trials contributing to improved outcomes in advanced cases (Table 6). Below is an overview of some of the most cutting-edge strategies.

#### 4.2.1. Intra-Arterial Chemotherapy

**Figure 7:**
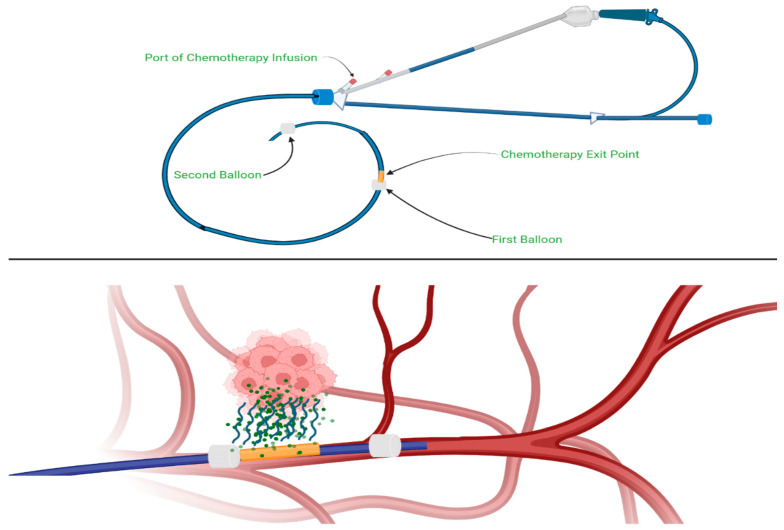
RenevoCath device structure and function to improve intra-arterial chemotherapy [5].

Currently, chemotherapy regimens such as FOLFIRINOX or gemcitabine combined with nab-paclitaxel are typically administered intravenously (IV). However, some clinical trials are investigating the outcomes of delivering standard-of-care treatments via alternative routes of administration. These alternative methods may prove effective as second- or third-line therapies, particularly in cases where IV chemotherapy yields poor results or when patients are refractory to certain standard regimens. Pancreatic cancer treatment has evolved significantly in recent years, with novel delivery methods, advanced procedures, and innovative clinical trials contributing to improved outcomes for patients with advanced disease. One such method, intra-arterial chemotherapy, involves delivering chemotherapeutic agents directly into the arteries supplying the tumor. In advanced pancreatic cancer, this typically requires infusion into both the celiac artery and the superior mesenteric artery (SMA), which makes the procedure technically challenging due to the need for dual arterial access [139]. Ishikawa et al. treated patients with metastatic pancreatic cancer using a combination therapy of gemcitabine, 5-fluorouracil (5-FU), and cisplatin mixed with angiotensin II, administered via intra-arterial injection through the right femoral artery [140]. Following this chemotherapy regimen, tumor marker levels decreased in 12 out of 20 patients, with a 6-month survival rate of 80% and a 1-year survival rate of 44.7%. Additionally, pain was reduced to a manageable level in all 20 patients, including the 6 who initially presented with back pain as a primary symptom. These findings suggest that using the right femoral artery as an access point may help overcome the challenges associated with dual-arterial infusion. Currently, a phase 3 clinical trial (NCT03257033) is actively recruiting patients. After receiving 4 months of radiation therapy and standard chemotherapy with gemcitabine and nab-paclitaxel, participants are randomized to receive either intra-arterial gemcitabine via dual-arterial infusion or continued standard intravenous chemotherapy with gemcitabine and nab-paclitaxel. In this trial, the RenevoCath device—a dual-balloon infusion catheter—is used to deliver gemcitabine intra-arterially. The use of RenevoCath may simplify and enhance the efficiency of dual-arterial chemotherapy delivery (Figure 7). Advancements in intra-arterial chemotherapy, whether through right femoral artery access or specialized devices like RenevoCath, could represent a significant breakthrough in the treatment of advanced pancreatic cancer, addressing a previously challenging therapeutic barrier.

#### 4.2.2. Nanoparticle and Liposomal Drug Delivery

pH-sensitive nanoparticles encapsulate chemotherapeutic agents such as gemcitabine and ERK inhibitors (ERKis), enabling selective drug release in the acidic microenvironment of tumor cells. This targeted delivery approach minimizes systemic toxicity and enhances therapeutic efficacy [141]. Nanoliposomal irinotecan, also known as MM-398 (Onivyde), is a liposomal formulation of irinotecan combined with 5-fluorouracil (5-FU) and leucovorin, designed to improve cytosolic drug delivery and reduce systemic toxicity. Onivyde has been evaluated in several clinical trials for the treatment of pancreatic cancer. The NAPOLI-1 trial, a phase 3 study, demonstrated that Onivyde plus 5-FU/leucovorin significantly improved overall survival (OS) in patients with gemcitabine-refractory metastatic pancreatic cancer. The median OS was 6.1 months in the Onivyde group compared to 4.2 months with 5-FU/leucovorin alone, a 1.9-month improvement (NCT01494506) [131,132]. These results led to the FDA approval of Onivyde in 2015 as a second-line treatment.

More recently, the FDA approved Onivyde for first-line treatment of metastatic pancreatic adenocarcinoma, based on the NAPOLI-3 trial. This trial expanded the use of Onivyde to the first-line setting, where it was used as part of the NALIRIFOX regimen (Onivyde + oxaliplatin + 5-FU/leucovorin). The NALIRIFOX regimen achieved a median OS of 11.1 months compared to gemcitabine plus nab-paclitaxel, demonstrating superior efficacy (NCT04083235). Onivyde’s liposomal delivery system improves tumor targeting and reduces systemic toxicity while synergizing with 5-FU to enhance cytotoxicity. Building on these findings, the PAN-HEROIC-1 study (NCT05074589) evaluated irinotecan hydrochloride liposome HR070803 in combination with 5-FU and leucovorin in patients with locally advanced or metastatic pancreatic ductal adenocarcinoma following prior gemcitabine-based therapy. This phase 3 trial demonstrated significant improvements in both overall survival (median 7.1 vs. 4.2 months) and progression-free survival (median 3.7 vs. 2.7 months) compared to placebo plus 5-FU/leucovorin [133]. Based on these results, HR070803 in combination with 5-FU/leucovorin received approval from the China National Medical Products Administration in January 2024 for second-line treatment of metastatic or LAPC, further expanding treatment options for this challenging disease.

#### 4.2.3. Irreversible Electroporation and Electrochemotherapy

Electroporation, particularly irreversible electroporation (IRE), uses high-voltage, low-energy electrical pulses to create nanopores in cell membranes, resulting in cell death while sparing surrounding critical structures. IRE has shown promise in treating unresectable pancreatic tumors, especially those located near major vascular structures [142]. IRE not only induces cell death through membrane disruption but also enhances the delivery and efficacy of chemotherapeutic and immunotherapeutic agents. Electrochemotherapy (ECT), a technique that combines IRE with chemotherapy, has shown encouraging results in the treatment of LAPC, with minimal adverse effects [143,144]. Preclinical studies have shown that IRE can safely synergize with chemotherapy agents such as bleomycin and FOLFIRINOX, significantly reducing tumor cell proliferation and improving treatment outcomes [143,145]. Moreover, IRE has demonstrated the potential to enhance immune responses by increasing the permeability of tumor cells to immunomodulatory agents, such as oncolytic viruses and anti-PD-1 checkpoint inhibitors. This permeability facilitates the activation of CD8^+^ T lymphocytes and the release of danger-associated molecular patterns (DAMPs) within the tumor microenvironment [142,146,147,148].

Several ongoing clinical trials are investigating the efficacy and safety of irreversible electroporation (IRE) in the treatment of pancreatic cancer. The DIRECT Registry study (NCT03899649) is a prospective trial evaluating the safety and effectiveness of IRE using the NanoKnife System in patients with Stage 3 PDAC. The study assesses IRE as a curative-intent tumor ablation method—either alone or in combination with surgical resection—following at least three months of induction chemotherapy. It compares IRE plus standard of care (SOC) treatment to SOC alone in a real-world setting [134]. Preliminary data from 87 IRE-treated patients demonstrate acceptable safety, with 30-day and 90-day mortality rates of 3.4% and 4.9%, respectively. Additionally, 24.1% of patients experienced IRE-related adverse events within 90 days post-procedure, with gastrointestinal complications being the most common [135]. These early findings suggest that IRE may be a feasible treatment option for appropriately selected patients with Stage 3 PDAC. Another ongoing trial (NCT06205849) is investigating the combination of intra-tumoral mitazalimab, a CD40 agonistic antibody, with IRE for patients with LAPC [149,150]. The combination of IRE with immunotherapy is an emerging approach for treating LAPC. The preclinical evidence suggests that IRE can enhance anti-tumor immune responses when combined with immunomodulatory agents [151]. In murine models of pancreatic cancer, IRE combined with immunotherapy has been shown to promote the selective infiltration of CD8^+^ T cells and significantly prolong survival [146]. A new clinical trial (NCT06205849) is investigating the combination of intra-tumoral mitazalimab, a CD40 agonistic antibody, with IRE for LAPC patients [136]. The rationale for combining irreversible electroporation (IRE) with CD40 agonism stems from preclinical studies demonstrating that IRE can induce immunogenic cell death, activate dendritic cells, and reduce stroma-induced immunosuppression [146,150]. Additionally, a phase 1 trial (NCT06378047) is investigating the safety and optimal dosing of IRE in combination with pembrolizumab immunotherapy for the treatment of pancreatic cancer. These studies aim to refine the role of IRE as either a standalone or combination therapy, with the goal of improving survival outcomes and QoL for patients with advanced pancreatic cancer.

#### 4.2.4. Ablation Procedures

Ablation techniques have shown promise in the treatment of advanced pancreatic cancer, although clinical data remain limited for some approaches.

##### Laparoscopic Microwave Ablation

Laparoscopic microwave ablation (MWA) has demonstrated technical feasibility and safety in small clinical studies [152]. In a 2007 study, Lygidakis et al. reported 100% technical success in 15 patients with LAPC treated with laparoscopic MWA, with partial tumor necrosis observed during follow-up [153]. Later, Carrafiello et al. compared laparoscopic and percutaneous MWA approaches in 10 patients, also reporting 100% technical success for both methods [154].

##### Radiofrequency Ablation

Radiofrequency ablation (RFA) has emerged as a promising minimally invasive technique for managing pancreatic cancer, particularly in unresectable cases. RFA uses focal thermal energy to induce cell death through coagulative necrosis and protein denaturation [155]. Recent studies have demonstrated RFA’s potential to reduce tumor progression, promote an abscopal effect, and induce significant remodeling of the tumor stroma and immune response [156]. RFA can be performed via open surgery, laparoscopic surgery, and more recently, endoscopic ultrasound-guided (EUS-guided) approaches [157]. EUS-guided RFA has gained attention as a minimally invasive option for treating pancreatic masses, offering real-time imaging and precise lesion targeting while minimizing damage to surrounding tissues [158]. A 2025 comprehensive review of EUS-guided RFA for pancreatic adenocarcinoma reported a pooled technical success rate of 100% in most studies, with a meta-analysis indicating a pooled adverse event rate of 22.6% [159].

While RFA appears to provide symptom palliation and may improve survival, larger prospective trials are needed to fully define its role in the overall management of pancreatic cancer [158,160]. This technique offers a dual benefit, direct tumor ablation and indirect enhancement of anti-tumor immunity, making it a potential adjunct to standard chemotherapy and immunotherapy regimens [156,161]. However, standardized guidelines remain lacking due to insufficient long-term data, and current applications are largely guided by individual physician experience and center-specific protocols [158,162]. Ongoing research continues to explore EUS-guided RFA for a variety of pancreatic lesions, including unresectable and LAPC, as well as pancreatic neuroendocrine tumors [159].

##### High-Intensity Focused Ultrasound

High-intensity focused ultrasound (HIFU) is a non-invasive therapeutic modality that uses focused ultrasound (US) energy to selectively ablate targeted lesions in the pancreas without damaging surrounding tissues [163]. HIFU has shown promise for pain relief and tumor control in patients with advanced pancreatic cancer, effectively alleviating cancer-related abdominal pain, reducing tumor mass, and improving QoL with minimal complications [163,164]. An ongoing randomized controlled clinical trial at University Hospital Bonn (DRKS00012367) in Germany is comparing HIFU combined with chemotherapy to chemotherapy alone in newly diagnosed inoperable pancreatic cancer patients, focusing on tumor volume, pain relief, and survival outcomes [137]. The collaborative consensus initiative between Chinese and European HIFU centers [138] aims to standardize US-guided HIFU protocols for pancreatic cancer treatment, focusing on patient selection, treatment endpoints (e.g., pain relief, tumor volume reduction), safety guidelines, and integration with systemic therapies like chemotherapy. This multinational effort seeks to harmonize clinical practices, building on pooled expertise to optimize the role of HIFU in palliative care for unresectable or metastatic pancreatic cancer, while addressing gaps in randomized evidence and advocating for its use alongside standard treatments to improve QoL and survival outcomes [138]. Furthermore, a phase 1/phase 2 multicenter trial (NCT06211933) is evaluating the safety and efficacy of intraoperative HIFU for locally advanced, unresectable pancreatic tumors. This study intends to assess the tolerance of intraoperative HIFU intervention on pancreatic lesions in phase 1 and evaluate its preliminary efficacy in phase 2. These studies aim to provide robust evidence on the role of HIFU in advanced pancreatic cancer treatment, addressing the need for larger, well-designed trials to establish its clinical utility in this aggressive disease.

##### Robotic Whipple Procedure

The robotic Whipple procedure is a minimally invasive surgical technique used to treat pancreatic cancer and other conditions affecting the pancreas, bile duct, and small intestine. It is an advanced form of the traditional Whipple surgery, also known as pancreaticoduodenectomy (PD) [165]. The PORTAL trial (NCT04400357) is a phase 3 multicenter randomized controlled trial comparing robotic vs. open pancreaticoduodenectomy. The trial’s primary endpoint is time to functional recovery postoperatively, with secondary assessments including postoperative morbidity, mortality, and perioperative costs. As of the current date, the recruitment status of this trial is unknown. A recent study on the “Four-Day Robotic Whipple” procedure demonstrated that patients undergoing robotic PD experienced significantly shorter hospital stays without increased complication or readmission rates compared to those undergoing open surgery. Notably, 40% of patients who underwent robotic PD had a length of stay of four days or fewer, representing a substantial improvement over traditional open techniques [166]. In addition, an ongoing clinical trial (NCT04763642) is comparing minimally invasive approaches, including laparoscopic and robotic pancreaticoduodenectomy, with open surgery for cancers of the pancreaticobiliary zone. The study focuses on perioperative outcomes and short-term survival metrics.

##### Stereotactic Body Radiotherapy

Stereotactic body radiotherapy (SBRT) is a highly precise form of external beam radiotherapy that delivers high doses of radiation to cancer cells while minimizing exposure to surrounding healthy tissue [167]. This advanced technique utilizes sophisticated image guidance and 3D or 4D imaging to pinpoint tumor location, enabling more accurate and effective treatment delivery [168]. In pancreatic cancer, SBRT has shown promise, particularly in patients with locally advanced or borderline resectable disease [169]. Recent clinical trials have demonstrated its efficacy in improving outcomes. For example, a study by Rudra et al. found that patients receiving high-dose SBRT (40–52 Gy) had significantly better survival rates than those treated with standard doses (30–35 Gy) [170]. The development of magnetic resonance-guided radiotherapy (MRgRT) has further enhanced SBRT precision, enabling real-time treatment adaptation and re-optimization prior to each fraction [170]. Additionally, ongoing research such as the study by Reddy et al. is exploring the combination of SBRT with immunotherapy to overcome treatment resistance in pancreatic cancer [171]. As SBRT technology continues to evolve, it holds the potential to improve therapeutic outcomes while reducing side effects and shortening treatment duration compared to conventional radiotherapy [172].

## 5. Conclusions

Advanced pancreatic cancer remains one of the most challenging malignancies to treat, with poor prognosis due to late detection, aggressive tumor biology, and resistance to conventional therapies. Standard therapies like FOLFIRINOX and gemcitabine/nab-paclitaxel offer modest benefits, especially in advanced stages. As a result, research is increasingly focused on novel approaches targeting the molecular, metabolic, and immune drivers of advanced PDAC. Ongoing trials are evaluating targeted therapies (e.g., KRAS, PARP, and mTOR inhibitors), immunotherapies (e.g., checkpoint inhibitors, CAR-T cells, and cancer vaccines), and local treatments like IRE, SBRT, and HIFU. New delivery methods such as pH-sensitive nanoparticles, nanoliposomal irinotecan (Onivyde), and intra-arterial chemotherapy using RenevoCath are enhancing drug targeting and reducing toxicity. Drug repurposing, involving agents like metformin, digoxin, simvastatin, auranofin, and parbendazole, presents a cost-effective strategy with encouraging preclinical results. Despite persistent challenges like chemoresistance and tumor heterogeneity, combination therapies integrating immune modulation, stromal targeting, and metabolic intervention are showing early promise. The evolving landscape of clinical trials, particularly those exploring cancer vaccines and ablation techniques, highlights the growing potential to improve outcomes. Continued innovation and well-designed studies remain critical to advancing care for patients with advanced pancreatic cancer.

## Figures and Tables

**Table 1:** Completed and active clinical trials for cytotoxic therapy in the treatment of advanced pancreatic cancer.

Drug	Action of Drug Used as Monotherapy or with Standard Care	Clinical Status	Phase	Clinical Studies and References
Olaparib	PARP inhibitor	Completed in 2019	Phase 3	NCT02184195 [38]
Lonsurf	Inhibits Thymidine Phosphorylase	Terminated in 2020	Phase 2	NCT02921737
Cisplatin + Gemcitabine	Inhibits DNA replication	Completed in 2002	Phase 3	(No clinical trial number; trial done outside the United States.) [41]
Capecitabine + Gemcitabine	Inhibits Expression of RANK/RANKL pathway proteins in HT29 Cell Line	Completed in 2009	Phase 2	(No clinical trial number; trial done outside the United States.) [42,43]
APG-1387 + Gemcitabine + Nab-paclitaxel	Inhibits apoptosis regulator proteins, IAPs	Recruiting	Phase 1/Phase 2	NCT04643405 [42]
Lonsurf + Gemcitabine + Nab-paclitaxel	Inhibits Thymidine Phosphorylase and interferes with DNA Synthesis	Completed in 2021	Phase 1	NCT04046887 [44]

**Table 2:** Completed and active clinical trials for targeted therapy in the treatment of advanced pancreatic cancer.

Drug	Action of Drug Used as Monotherapy or with Standard Care	Clinical Status	Phase	Clinical Studies and References
Adagrasib	KRAS inhibitor	Recruiting	Phase 1/Phase 2	NCT03785249 [50]
Adagrasib	KRAS inhibitor	Recruiting	Phase 1b	NCT05634525
Sotorasib	KRAS inhibitor	Active, Not Recruiting	Phase 1/Phase 2	NCT03600883 [51]
RMC-6236	Pan-RAS inhibitor	Recruiting	Phase 1	NCT05379985
Minnelide	Interferes with Hyaluronan Synthesis	Completed in 2019	Phase 2	NCT03117920 [52]
Marimastat	A novel matrix metalloproteinase inhibitor	Completed in 2001	Phase 2	(No clinical trial number; trial done outside the United States.) [53,54]
Sirolimus	mTOR inhibitor	Unknown	Phase 2	NCT03662412
QTX3034 or QTX3034 + Cetuximab	KRAS inhibitor or KRAS inhibitor + Anti-EGFR antibody	Recruiting	Phase 1	NCT06227377 [55]
HRS-4642 + Adebelimab	KRAS inhibitor + PD-L1 inhibitor	Recruiting	Phase 1/Phase 2	NCT06427239
HRS-4642 + ADC	KRAS inhibitor	Not yet recruiting	Phase 2	NCT06547736
HRS-4642 + Nimotuzumab	KRAS inhibitor + EGFR inhibitor	Not yet recruiting	Phase 1/Phase 2	NCT06773130
HRS-4642 + Nimotuzumab + Gemcitabine + Nab-paclitaxel	KRAS inhibitor + EGFR inhibitor + Chemotherapy	Not yet recruiting	Phase 2	NCT06770452
BI 1823911 or BI 1823911 + BI 1701963	SOS1 inhibitor or SOS1 inhibitor + SHP2 inhibitor	Active, not recruiting	Phase 1	NCT04973163 [56]
Nimotuzumab + Gemcitabine	EGFR inhibitor + Chemotherapy	Completed in 2013	Phase 2/Phase 3	NCT00561990 [57]
Nimotuzumab + Gemcitabine	EGFR inhibitor + Chemotherapy	Completed in 2021	Phase 3	NCT02395016 [58]
Nimotuzumab + NALIRIFOX	EGFR inhibitor + Chemotherapy	Active, not recruiting	Phase 2	NCT06429904
Nimotuzumab + gemcitabine + nab-paclitaxel	EGFR inhibitor + Chemotherapy	Recruiting	Phase 2	NCT06404840
Zenocutuzumab (MCLA-128)	Her 2 and Her 3 bispecific antibody	Active, not recruiting		NCT02912949 [59]
MK-0646 + gemcitabine or MK-0646 + gemcitabine + Erlotinib	Anti-IGF-1R antibody + Chemotherapy or Anti-IGF-1R antibody + Chemotherapy + EGFR inhibitor	Completed 2020	Phase 1/Phase 2	NCT00769483 [60,61]
Vandetanib + Gemcitabine	RET inhibitor + Chemotherapy	Completed in 2017	Phase 2	(No clinical trial number; trial done outside the United States.) [62]
Axitinib + Gemcitabine	VEGFR inhibitor + Chemotherapy	Completed in 2008	Phase 2	NCT00219557 [63]
Axitinib + Gemcitabine	VEGFR inhibitor+ Chemotherapy	Completed in 2009	Phase 3	NCT00471146 [64]
PEGPH20 + Gemcitabine + Nab-paclitaxel	Targets and breaks down hyaluronan + Chemotherapy	Terminated	Phase 3	NCT02715804 [65]
PEGPH20 + Gemcitabine + Nab-paclitaxel	Targets and breaks down hyaluronan + Chemotherapy	Active, not recruiting	Not applicable	NCT02921022

**Table 3:** Completed and active clinical trials for metabolic inhibitors in the treatment of advanced pancreatic cancer.

Drug	Action of Drug Used as Monotherapy or with Standard Care	Clinical Status	Phase	Clinical Studies and References
Telaglenastat hydrochloride (CB-839 HCl)	Glutaminase inhibitor	Completed 2019	Phase 1	NCT02071862 [99]
Telaglenastat hydrochloride (CB-839 HCl)	Glutaminase inhibitor	Active, not recruiting	Phase 2	NCT03872427
Devimistat (CPI-613) + mFOLFIRINOX	Mitochondrial metabolism inhibitor targeting both PDH and α-KGDH	Completed 2023	Phase 1	NCT01835041 [100]
Devimistat (CPI-613) + mFOLFIRINOX	Mitochondrial metabolism inhibitor targeting both PDH and α-KGDH	Completed 2022	Phase 3	NCT03504423 [101]
Devimistat (CPI-613) + chemoradiation	Mitochondrial metabolism inhibitor targeting both PDH and α-KGDH	Recruiting	Phase 1	NCT05325281
Devimistat (CPI-613) + Hydroxychloroquine + 5-fluorouracil or Gemcitabine	Mitochondrial metabolism inhibitor targeting both PDH and α-KGDH + Antimalarial + Chemotherapy	Active	Phase 2	NCT05733000
Devimistat (CPI-613) + Modified FOLFIRINOX	Mitochondrial metabolism inhibitor targeting both PDH and α-KGDH + Chemotherapy	Withdrawn	Phase 1/Phase 2	NCT05926206
AG-270	MAT2A inhibitor	Terminated	Phase 1	NCT03435250 [102]
Racemetyrosine (SM-88) + methoxsalen, phenytoin, and sirolimus (MPS)	Disrupts tyrosine-mediated metabolic pathways + Other agents	Terminated	Phase 2/Phase 3	NCT03512756
Racemetyrosine (SM-88) + mFOLFIRINOX + pamrevlumab + gemcitabine + nab-paclitaxel + canakinumab + spartalizumab	Disrupts tyrosine-mediated metabolic pathways + Chemotherapy + Other targeted agents	Active, Not Recruiting	Phase 3	NCT04229004

**Table 4:** Completed and active clinical trials for immunotherapy in the treatment of advanced pancreatic cancer.

Drug	Action of Drug Used as Monotherapy or with Standard Care	Clinical Status	Phase	Clinical Studies and References
Pembrolizumab + XL888	PD-1 Inhibitor + Blocks enzymes needed for tumor growth	Completed in 2021	Phase 1	NCT03095781
Pembrolizumab + BCA101	PD-1 Inhibitor + Targets EGFR and TGFβ	Recruiting	Phase 1	NCT04429542
Pembrolizumab + NGM831	PD-1 Inhibitor + ILT3 antagonist antibody	Active, Not Recruiting	Phase 1	NCT05215574
Pembrolizumab + Lenvatinib	PD-1 Inhibitor + VEGFR inhibitor	Active, Not Recruiting	Phase 1	NCT05273554
Pembrolizumab + SD-101	PD-1 Inhibitor + TLR 9 agonist	Recruiting	Phase 1	NCT05607953
Pembrolizumab + NT-17	PD-1 Inhibitor + Promotes T-cell development	Active, Not Recruiting	Phase 1/Phase 2	NCT04332653
Pembrolizumab + ONC-392	PD-1 Inhibitor + A humanized anti-CTLA4 IgG1 monoclonal antibody	Recruiting	Phase 1/Phase 2	NCT04140526 [111,112,113,114,115,116]
Durvalumab + Tazemetostat	PDL-1 Inhibitor + EZH2 inhibitor	Recruiting	Phase 2	NCT04705818
Pembrolizumab + Durvalumab + Ipilimumab	PD-1 Inhibitor + PDL-1 Inhibitor + CTLA4 Inhibitor	Recruiting	Phase 1/Phase 2	NCT05187338
U87 CART-T cells	Engineered receptors that bind to tumor antigens	Recruiting	Phase 1	NCT05605197
IM92 CAR-T cells	Engineered receptors that bind to tumor antigens	Unknown	Early Phase 1	NCT05275062
IM96 CAR-T cells	Engineered receptors that bind to tumor antigens	Unknown	Early Phase 1	NCT05287165
Spevatamig (PT886)	Antibody targeting Claudin 18.2 (CLDN18.2) and CD47	Unknown	Phase 1	[119]
Spevatamig (PT886)	Antibody targeting Claudin 18.2 (CLDN18.2) and CD47	Active	Phase 1/Phase 2	NCT05482893
GVAX vaccine + Ipilimumab + FOLFIRINOX	Stimulates anti-tumor immune responses + Blocks T-cell inhibitory signals + Chemotherapy	Completed 2019	Phase 2	NCT01896869
GVAX vaccine + Pembrolizumab + Cyclophosphamide + Stereotactic Body Radiation Therapy (SBRT)	Stimulates anti-tumor immune responses + Blocks T-cell negative signals + chemotherapy + radiation	Completed 2022	Phase 2	NCT02648282
GV1001 vaccine + Gemcitabine + Capecitabine	Telomerase peptide vaccine aiding in an immune system response + Chemotherapy	Completed 2013	Phase 3	NCT00425360
iNeo-Vac-P01	Peptide noeoantigen vaccine	Completed 2021	Phase 1	NCT03645148
Autogene Cevumeran + Atezolizumab + mFOLFIRINOX	mRNA Vaccine + PD-L1 inhibitor + Chemotherapy	Recruiting	Phase 2	NCT05968326

**Table 5:** Complete and active clinical/preclinical trials for repurposed drugs in the treatment of advanced pancreatic cancer.

Drug	Action of Drug Used as Monotherapy or with Standard Care	Clinical Status	Phase	Clinical Studies and References
Metformin + Digoxin + Simvastatin	Targets PDX1 and BIRC5	Recruiting	Phase 1	NCT03889795
Auranofin	Inhibits Txnrd1 and HIF-1α	Not Applicable (in-vivo study)	Not Applicable	(Preclinical) [128]
Parbendazole	Fosters apoptosis	Not Applicable (in-vitro study)	Not Applicable	(Preclinical) [130]

**Table 6:** Complete and active clinical trials for novel delivery methods and advanced procedures in the treatment of advanced pancreatic cancer.

Delivery Method/Procedure	Device Used/Vehicle Used or with Standard Care	Clinical Status	Phase	Clinical Studies and References
Intraarterial chemotherapy with gemcitabine	RenovoCath	Recruiting	Phase 3	NCT03257033
Nanoliposomal irinotecan (Onyvide) + 5-FU/leucovorin	Nanoparticle + Chemotherapy	Completed 2015	Phase 3	NCT01494506 [131,132]
Nanoliposomal irinotecan + oxaliplatin + 5-FU/leucovorin (NALIRIFOX)	Nanoparticle + Chemotherapy	Completed 2025	Phase 3	NCT04083235
Irinotecan liposomal hydrochloride (HR070803) + 5-FU/leucovorin	Liposome + Chemotherapy	Completed 2022	Phase 3	NCT05074589 [133]
IRE +SOC	IRE	Active, not recruiting	Observational	NCT03899649 [134,135]
1IRE+ Intra-tumoral mitazalimab, a CD40 agonistic antibody	IRE	Recruiting	Phase 1	NCT06205849 [136]
IRE + Pembrolizumab	IRE	Recruiting	Phase 1	NCT06378047
HIFU + Standard chemotherapy	HIFU	Recruiting	Phase 1/Phase 2	DRKS00012367 [137]
US-guided HIFU	HIFU	Ongoing	No information available	[138]
HIFU	HIFU	Recruiting	Phase 1/Phase 2	NCT06211933
Robotic pancreaticoduodenectomy	Robotic Whipple Therapy	Unknown Status	Not applicable	NCT04400357
Miniinvasive pancreaticoduodenectomy	Robotic Whipple Therapy	Active, not recruiting	Not applicable	NCT04763642

## Data Availability

No new data were created or analyzed in this review paper. Data sharing is not applicable to this article.
